# Bivariate One Strain Many Compounds Designs Expand the Secondary Metabolite Production Space in *Corallococcus coralloides*

**DOI:** 10.3390/microorganisms11102592

**Published:** 2023-10-20

**Authors:** Anton Lindig, Jenny Schwarz, Georg Hubmann, Katrin Rosenthal, Stephan Lütz

**Affiliations:** 1 Department of Biochemical and Chemical Engineering, TU Dortmund University, Emil-Figge-Straße 66, 44227 Dortmund, Germany; 2 School of Science, Constructor University, 28759 Bremen, Germany; krosenthal@constructor.university

**Keywords:** myxobacteria, secondary metabolites, multifactorial OSMAC, screening method

## Abstract

The scarcely investigated myxobacterium *Corallococcus coralloides* holds a large genome containing many uncharacterized biosynthetic gene clusters (BGCs) that potentially encode the synthesis of entirely new natural products. Despite its promising genomic potential, suitable cultivation conditions have not yet been found to activate the synthesis of new secondary metabolites (SMs). Finding the right cultivation conditions to activate BGCs in the genome remains a major bottleneck, and its full biosynthetic potential has so far not been determined. We therefore applied a bivariate “one strain many compounds” (OSMAC) approach, using a combination of two elicitor changes at once, for the activation of BGCs and concomitant SM production by *C. coralloides*. The screening was carried out in Duetz-System 24-well plates, applying univariate and bivariate OSMAC conditions. We combined biotic additives and organic solvents with a complex growth medium for univariate conditions and with minimal medium for bivariate conditions. The success in the activation of BGCs was evaluated by determining the number of new mass features detected in the respective extracts. We found synergistic effects in the bivariate OSMAC designs, evidenced by the detection of completely new mass features in the bivariate OSMAC experiments, which were not detected in the univariate OSMAC designs with only one elicitor. Overall, the bivariate OSMAC screening led to 55 new mass features, which were not detected in the univariate OSMAC design. Molecular networks revealed that these new mass features embody potential novel natural compounds and chemical derivatives like the *N*-acyl fatty amine *N*-pentyloctadecanamide and possibly sulfur-containing natural products. Hence, the presence of multiple elicitors in the bivariate OSMAC designs successfully activated the biosynthetic potential in *C. coralloides*. We propose bivariate OSMAC designs with a complex combination of elicitors as a straightforward strategy to robustly expand the SM space of microorganisms with large genomes.

## 1. Introduction

Bacteria are among the most promising sources of new bioactive compounds such as antibiotics. However, only a small percentage of microorganisms have been investigated for their ability to synthesize secondary metabolites. It is estimated that only between 1 and 15% of the microbial world has been cultured until today [1]; hence, the majority of bacteria remain unknown and are referred to as microbial dark matter with unknown biosynthetic potential [2]. In addition, known and sequenced bacteria still show huge uncharacterized biosynthetic potential in their genomes that has not been related to the production of any secondary metabolites (SMs) yet. These regions are often arranged in biosynthetic gene clusters (BGCs) in the microbial genome and outnumber the known SMs by a factor of 5 to 10 [3,4,5]. Known BGCs can be assigned to the different classes of SMs, e.g., nonribosomal peptides, terpenes and polyketides [6]. A positive correlation between genome size and the number of BGCs has been found [7]. Thus, bacteria with large genomes generally possess more BGCs and have been frequently reported as a source of new antibiotics.

Bacteria with extremely large genomes were found among the Gram-positive actinobacteria and the Gram-negative proteobacteria. Within the latter phylum, myxobacteria are well known for their predation of Gram-positive bacteria such as Bacillus strains through antibiotic compounds [8]. *Bacillus subtilis* is a known prey to *Myxococcus xanthus* and has also developed a defense mechanism by producing bacillaene—a well-known and characterized antibiotic [9]. Myxobacterial SMs with antibiotic activity are rather new and mainly comprise gulmirecins, myxopyronins and corallopyronins [10]. However, further investigations are necessary to fully exploit the entire biosynthetic potential of myxobacteria and actinobacteria, as a rich source of various chemically distinguished SMs [11]. According to Livingstone et al., *Corallococcus* species are potent sources of many new compounds due to their individual predatory activity that require a diversified biochemistry [12]. *Corallococcus* species are commonly found in soil and have attracted increased interest due to the detected antibacterial compounds corallopyronins A–C, corallozine A and coralmycins A and B [13]. *Corallococcus* species have a large genome and many unexplored genomic features, yet finding the right cultivation conditions to activate BGCs in their genomes remains a major bottleneck [14]. So far, only three natural products have been isolated from *Corallococcus coralloides* cultures: corallorazines [15], coralmycins [16] and corallopyronin [17]. There are only a few studies that have investigated *C. coralloides*, and no new SM compounds have so far been reported for this specific strain, despite its genome carrying BGCs potentially coding for SMs [18]. Most studies of *C. coralloides* have been performed as in silico genomics [12,18,19,20].

In the present study, the SM production space of the myxobacterium *C. coralloides* DSM2259 was investigated with a multifactorial OSMAC design in order to activate its full biosynthetic potential. In our previous study, *C. coralloides* was included in a multitude of OSMAC screening conditions. However, none of the 12 known BGCs and other predicted genomic features were activated using a single elicitor [14]. We assumed that the exposure to a single stimulus was not sufficient for the activation of its BGCs. To better exploit this largely unknown biosynthetic potential, we exposed *C. coralloides* to multiple elicitors in a single OSMAC design, providing a multitude of stimuli at once for the robust generation of new SM mass features (MFs). In our novel bivariate OSMAC approach, we tested and combined a minimal medium and a complex medium with biotic additives and organic solvents in cultures of *C. coralloides*. Based on the appearance of new MFs in LC-MS/MS measurements of the extracted supernatant samples, we compared the efficiency of SM production in bi- and univariate conditions in terms of the generation of new MFs and condition-specific compounds. Bivariate condition-specific MFs were further investigated in terms of their possible chemical properties and molecular structure with feature-based molecular networks.

## 2. Materials and Methods

### 2.1. Assessment of the Biosynthetic Potential

The bioinformatics genome analysis was conducted using the online tool antiSMASH bacterial version 6.1 [21]. The genome data of *C. coralloides* DSM2259 were obtained from NCBI (accession number: CP003389). Detection strictness was set to relaxed mode. Extra features were kept in default mode (KnownClusterBlast, ActiveSiteFinder and SubClusterBlast: ON, ClusterBlast, ClusterPfamAnalysis and Pfam-based GO term annotation: OFF). The results are listed in Table S1.

### 2.2. Bivariate OSMAC Experiments

The three utilized media were SP medium, M9 medium and MD1 medium.

SP medium: raffinose × 5 H_2_O: 1.18 g/L, sucrose: 1.0 g/L, galactose: 1.0 g/L, soluble starch: 5.0 g/L, Bacto^®^ casitone: 2.5 g/L, MgSO_4_ × 7 H_2_O: 0.5 g/L, K_2_HPO_4_: 0.25 g/L, final pH = 7.4.

M9 medium: 0.25 g/L MgSO_4_ × 7 H_2_O, 0.015 g/L CaCl_2_ × 2 H_2_O, 1 mL/L 1 M thiamine-HCl × 2 H_2_O, 2.1 g/L D(+) glucose, 20 mg/L L-proline, 100 mL/L salt solution (60 g/L Na_2_HPO_4_, 30 g/L KH_2_PO_4_, 10 g/L NH_4_Cl, 5 g/L NaCl), pH 7.4.

MD1 medium: 3.0 g/L casitone, 0.7 g/L CaCl_2_ × 2 H_2_O, 2.0 g/L MgSO_4_ × 7 H_2_O, 1 mL/L trace element solution (TES) SL4, 0.5 mg/L vitamin B12, pH 7.0 with a composition of TES SL4: 0.5 g/L EDTA, 0.2 g/L FeSO_4_ × 7 H_2_O, 0.01 g/L ZnSO_4_ × 7 H_2_O, 0.003 g/L MnCl_2_ × 4 H_2_O, 0.03 g/L H_3_BO_3_, 0.02 g/L CoCl_2_ × 6 H_2_O, 0.001 g/L CuCl_2_ × 2 H_2_O, 0.002 g/L NiCl_2_ × 6 H_2_O, 0.003 g/L Na_2_MoO_4_ × 2 H_2_O.

A list of chemical suppliers is provided in Table S2.

The investigated additives were ethanol (EtOH, 1% *v*/*v*), toluene (Tol, 1% *v*/*v*), autoclaved cell pellets (P, 3% *v*/*v*) and autoclaved supernatants (Sup, 10% *v*/*v*) of *Bacillus amyloliquefaciens* DSM7 (Ba) and *Streptomyces griseochromogenes* DSM40499 (Sg).

Duetz-System 24-well plates (Beckman Coulter, Aachen, Germany) were used in the bivariate OSMAC experiments. Each plate contained triplicates (=three wells) of the control group (*C. coralloides* on SP medium), one blank well with non-inoculated SP medium (contamination control), duplicates of the univariate factors (media and additives, 4 factors per plate, 8 wells in total) and triplicates of the bivariate setups (4 bivariate setups per plate, 12 wells in total).

Three different plate setups were used: one plate setup for pellet additives, one plate setup for supernatant additives and one plate setup for solvent additives. All plate setups were used in duplicate. In total, 6 SD plates were cultured.

The plates were used with a total liquid volume of 3 mL. The initial OD600 was set to 0.1 with precultured *C. coralloides* (24 h, 30 °C, 200 rpm) for inoculation. The inoculated plates were incubated on a rotary shaker with 2.5 cm deflection at 200 rpm at 30 °C for 140 h. The inoculation scheme and the plate setup can be found in the Supporting Information in Figures S1–S3.

### 2.3. Preparation of Biotic Additives

Biotic additives were generated from an *B. amyloliquefaciens* culture grown on NB medium (peptone from meat: 5.0 g/L, meat extract: 3.0 g/L, final pH = 7.0) and an *S. griseochromogenes* culture grown on GYM medium (glucose 5 × H_2_O: 4.4 g/L, yeast extract: 4.0 g/L, malt extract: 10.0 g/L, final pH = 7.2). Both strains were cultivated for 22 h at 30 °C and 150 rpm.

Sterile filtered supernatant: A total of 10 mL was transferred to a falcon tube in a sterile environment, centrifuged and sterile filtered into a fresh falcon tube. Separate stocks for the *B. amyloliquefaciens* supernatant and for the *S. griseochromogenes* supernatant were prepared and used as additives according to the plate setup in Table S1.

Cell pellet: A total of 10 mL of the culture was autoclaved, transferred to a falcon tube in a sterile environment and centrifuged afterwards. The supernatant was removed from the centrifuged falcon tube and discarded. The remaining autoclaved cell pellet was resuspended in 1 mL of culture medium.

### 2.4. Extraction and Sample Preparation

Cells were separated from the supernatant via centrifugation of 1 mL in Eppendorf cups at 13,000 rpm for 10 min. A total of 500 µL of the supernatant was transferred to separate glass Pyrex^®^ tubes for preparation of the metabolite extracts. An equivalent volume of 500 µL ethyl acetate was added to each tube and extracted using a multirotator (Grant-bio PTR-60, Grant Instruments Ltd., Shepreth, UK) for 2 min at 50 rpm with a change in direction every 3 s for better mixing of the 2-phase system. This was repeated once with removal of the organic phase and addition of fresh solvent in between each round. The ethyl acetate extract was collected in LC glass vials and evaporated to dryness in a Concentrator Plus vacuum centrifuge by Eppendorf. The vacuum–high vapor (V-HV)-mode was used at 30 °C for 40 min. A total of 75 µL of MS-grade methanol was added to each dry extract to dissolve the extracted compounds.

### 2.5. LC-MS Measurement

The sample extracts were analyzed using a 1290 Infinity II UHPLC from Agilent Technologies (Agilent Technologies, Santa Clara, CA, USA) coupled with a compact electrospray ionization–quadrupole–time-of-flight (ESI-Q-ToF) mass spectrometer system from Bruker (Bruker, Billerica, MA, USA). Acetonitrile (solvent A) and 0.1% formic acid in water (solvent B) were used as solvents with a flow rate of 0.4 mL/min. The injected sample volume was 2 µL. The following UHPLC method was employed: 0–10 min 5–98% solvent A, 10–15 min 98% solvent A isocratic, 15–17 min 98–5% solvent A, 17–20 min 5% solvent A (Solvent B was added accordingly to complete the solvent mixture) at a temperature of 40 °C with a C18-column (100 × 2.6 mm NucleoShell RP18, Macherey&Nagel, Düren, Germany). The mass spectrometer, comprising an ESI ion source (nebulizing gas pressure: 4 bar, drying gas flow: 12 L/min, drying temperature: 220 °C, capillary voltage: 4500 V, end plate offset: 500 V) and ToF analyzer (100–1000 *m*/*z*, scan rate: 20 Hz), was used with data-dependent auto-MS/MS acquisitions using an MS-MS/MS cycle time of 0.5 s in positive mode.

### 2.6. Mass Feature Detection and Structure Elucidation

The mass feature data analysis was performed using Data Analysis 4.4 (Bruker, Bremen, Germany) and MZmine 2.35 [22]. The steps and settings for raw data processing and peak list processing in MZmine are provided in Tables S3 and S4. To generate the list of MFs, the detected MS ions with their retention time (rt) and *m*/*z* pairs were grouped (rt_*m*/*z*). MFs were only considered for further evaluation steps if they satisfied the following criteria: (i) abundance >1000, (ii) available MS/MS-fragmentation, (iii) no detection in additive or medium samples and (iv) appearance in at least 2 of 3 extracts of a triplicate experiment. To identify new MFs specific to univariate screenings like the minimal medium, solvent and biotic additive, MFs from the control group sample (*C. coralloides* grown on SP medium) were removed from all MFs detected in the univariate sample extracts. For bivariate MFs, the MFs from the control group and from the univariate samples were subtracted from all detected MFs in the bivariate sample. Comprehensive lists of all MFs in the bivariate samples are provided in Table S7. MF in silico annotations were performed within the software SIRIUS 5.62 using precursor mass, fragmentation mass and retention time in default mode. A feature-based molecular network was created for the bivariate MFs and their associated MFs using the online workflow at Global Natural Products Social Molecular Networking (GNPS) in default mode (Available online: https://gnps.ucsd.edu/ (accessed on 13 September 2022)) [23]. Briefly, MS/MS container files and feature quantification tables were exported using MZmine. For the molecular network, a precursor ion mass tolerance and fragment ion mass tolerance of 0.02 Da was used. Further network criteria were set as follows: minimum pairs cosine of 0.7; minimum matched fragment ions of 6; maximum shift between precursors of 500 Da; and a network TopK of 10. The fragmentation spectra in the network were subsequently compared against GNPS spectral libraries. Only matches with a score > 0.6 and at least 6 matched peaks were further considered. Levels of confidence for annotation were assigned according to the recommendation of Sumner et al. [24]. Briefly, Tier 2 annotations were obtained for MFs with GNPS spectral library hits (Figure S4). MFs were annotated as tier 3 when a putative structure matched the precursor mass and the MS/MS fragmentation during in silico analysis and resulted in the best hit. Tier 4 relates to MFs with only a precursor mass and a unique retention time. Cytoscape version 3.6.1 was used to visually display the molecular network [25]. The abundance ratios of each node in the molecular network were presented in pie charts showing the highest observed intensity of each ion in the univariate and bivariate conditions. Some nodes in molecular families were labeled with their name, MFs (rt_*m*/*z*) and annotation confidence level (if available). All nodes represent the parent peaks detected as [M + H] + ions, unless indicated otherwise. Molecular structures were generated using ChemDraw 20.0.

## 3. Results and Discussion

A high number of BGCs were detected in the genome of *C. coralloides* DSM2259 (Figure S1); however, none of the predicted compounds have been detected until now [14]. Thus, the activation of the biosynthetic potential requires a novel approach. We chose to combine two stimuli at once to test whether synergistic effects in the SM production of *C. coralloides* can be observed using bivariate OSMAC approaches. The mass features (MFs), detected in sample extracts after OSMAC cultivation, were used as a proxy of the generated SMs in terms of their total number and their condition specificity in various OSMAC conditions.

### 3.1. The Biosynthetic Potential of Corallococcus coralloides DSM2259

*C. coralloides* DSM2259 was selected for this study, because this strain is a rather unknown bacterium with a large and fully sequenced genome. Its genome harbors many gene clusters that code for potential natural products. Many of these compounds have not yet been identified.

**Figure 1:**
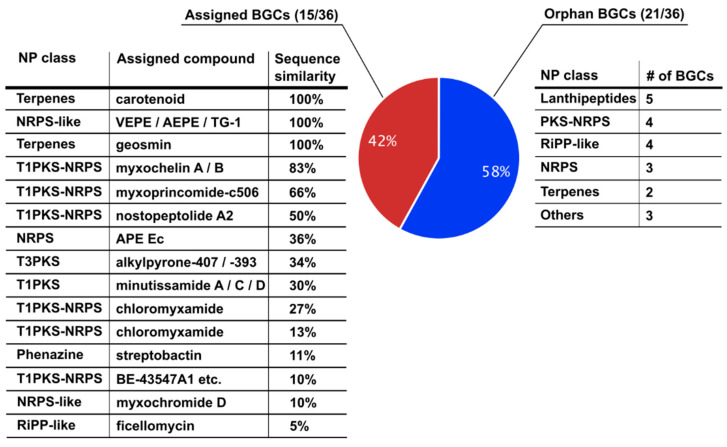
Genome mining of *C. coralloides* DSM2259. The pie chart presents the ratio of assigned and orphan BGCs identified on the genome of *C. coralloides* DSM2259 using the genome mining tool antiSMASH 6.1. The genome sequence was obtained from NCBI with the code CP003389.1. Genome mining analysis was performed in relaxed mode. The assigned BGCs include the natural product (NP) class, the assigned compound and the sequence similarity to an annotated BGC in the BGC database. The identification of orphan BGCs includes their NP class and the number of detected BGCs in the entire genome. Detected NP classes are terpenes, nonribosomal peptides (NRPS), type-I (T1) and type-III polyketides (PKS), phenazine, ribosomally synthesized and post-translationally modified peptides (RiPP), and lanthipeptides.

*C. coralloides* possesses a large genome of 10.08 Mbp and 36 BGCs coding for potential SMs, and accordingly, has large biosynthetic potential [14]. We analyzed the genomic sequence of DSM2259 (NCBI accession # CP003389) using antiSMASH bacterial version 6.1 and found that 13.4% of the *C. coralloides* genome was related to the biosynthesis of SMs. In total, the in silico analysis yielded 33 BGCs assigned to the biosynthesis of different natural product classes. Figure 1 summarizes the genome analysis. Among them were polyketides (PKS), nonribosomal peptides (NRPS), terpenes and lanthipeptides. Putative products were assigned to 15 BGCs by antiSMASH, but none of them have so far been reported for this strain. A detailed list of the assigned classes and putative SM products is presented in Table S1. The BGCs assigned to geosmin, the vinyl-/alkyl ether lipids—1-O-(13-methyl-1-Z-tetradecenyl)-2-O-(13-methyltetradecanoyl)-glycero-3-phosphatidylethanolamine (VEPE)/1-O-(13-methyltetradecyl)-2-O-(13-methyltetradecanoyl)glycero-3-phosphatidylethanolamine (AEPE)—and a carotenoid display 100% sequence similarity to the entries from the Minimum Information about a Biosynthetic Gene Cluster (MIBiG) database. Geosmin, the volatile terpenoid responsible for the earthy smell of several bacterial cultures, is also produced by different myxobacterial strains [26]. The ether lipids VEPE and AEPE have been reported to be produced by other myxobacteria as signal molecules for the differentiation part of the myxobacterial life cycle [27]. The prediction of a carotenoid is also not surprising since *Corallococcus* sp. is known for the formation of orange fruiting bodies [13]. Additionally, 83% similarity to the myxochelin A/B BGC and 66% similarity to the myxoprincomide BGC were detected. The other eleven assigned BGCs possess less than 50% similarity to the MIBiG database entries. Twenty-one BGCs could not be assigned to any BGCs in the MIBiG database and are, therefore, referred to as orphan BGCs [12].

The large number of orphan BGCs in *C. coralloides* DSM2259 require novel strategies to fully discover and characterize their biosynthetic potential.

### 3.2. Discovery of New Secondary Metabolite Mass Features in Uni- and Bivariate OSMAC Screening

In our previous study, we found that the metabolic response in the production of SMs of *C. coralloides* to various OSMAC stimuli was rather low despite its high biosynthetic potential [14]. This observation motivated us to modify the OSMAC approach with a multifactorial OSMAC design. To establish these OSMAC designs, we first combined two OSMAC stimuli in one experiment and evaluated the number of new MFs, as a proxy of newly generated SMs by *C. coralloides*. We evaluated the novel bivariate OSMAC approach in terms of the total number and the condition specificity of generated MFs. The new MFs in uni- and bivariate OSMAC cultivations were defined as not being produced under control conditions. As a control and reference, we cultured *C. coralloides* in SP medium without any additives and characterized the generated MFs in an LC-MS/MS analysis of sample extracts. Only the MFs that were not detected in the control but in the uni- or bivariate extracts were termed new MFs. We did not investigate further the intensity of new MFs in response to the OSMAC conditions.

**Figure 2:**
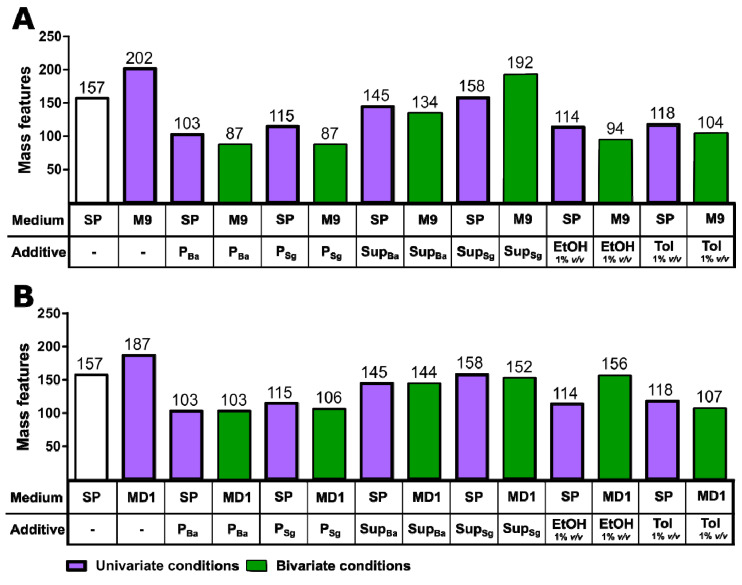
Number of mass features in univariate and bivariate conditions. The bar charts show the total number of mass features detected in the control condition (SP-medium, white bar), the univariate OSMAC (minimal medium, SP-medium with biotic or chemical additive, purple bars) and bivariate OSMAC (minimal medium with additive, green bars). The total number of new MFs that satisfied the following criteria was determined: (i) abundance > 1000, (ii) available MS/MS-fragmentation, (iii) no detection in additive or medium samples and (iv) appearance in at least 2 of 3 extracts of the triplicate experiment obtained in M9 minimal medium (**A**) and MD1-medium (**B**). Ethanol (EtOH) and toluene (TOL) were used as solvent additives and added to the OSMAC cultivations at a volume equivalent of 1%. The biotic additives were obtained from cultivations of *B. amyloliquefaciens* (Ba) and *S. griseochromogenes* (Sg). A total of 300 µL of the culture supernatant (Sup) or 100 µL of the obtained cell pellets (P) was added to the uni- and bivariate OSMAC experiments.

Figure 2A shows that a large number of new MFs were detected in uni- and in bivariate OSMAC setups with both the minimal medium M9 and the nutrient-rich MD1 medium. The addition of bacterial supernatants resulted in a higher number of new MFs than the addition of bacterial cell pellets. A greater difference can be seen in the minimal medium cultures. In *C. coralloides* cultures grown on M9 medium, 87 new MFs were detected, while in cultures in which the supernatant of Ba or Sg was added, 134 and 192 new MFs were detected, respectively. The addition of cell pellets from Ba or Sg in cultures grown in MD1 resulted in the formation of 103 and 106 new MFs, while 144 and 152 new MFs were detected in cultures to which the supernatant of Ba or Sg was added. The addition of organic solvents also led to a similar number of new mass features in comparison to the biotic additives. Moreover, 45 unique new MFs were detected in the bivariate conditions with M9 medium combined with supernatant cultures of Ba or Sg, and 5 MFs were detected exclusively in MD1 medium with organic solvent added.

In the literature, biotic additives have been reported to successfully induce or increase antibiotic production [28]. In our study, the addition of additives regardless of the type of medium was very effective for the synthesis of new MFs, indicating the production of new secondary metabolites. Taken together, all six bivariate setups resulted in more than 80 new MFs each. It has to be noted that the displayed new MFs were curated so that MFs originating from the medium or additive background were filtered out. MFs originally detected in univariate culture conditions can still be among the MFs detected in the bivariate extracts. However, new condition-specific MFs in bivariate samples were detected that were not observed in univariate cultures.

We showed that the bivariate OSMAC approach displayed synergistic properties when combining minimal or nutrient-rich media with autoclaved cell pellets, supernatants or organic solvents such as ethanol and toluene. It has been reported that the addition of toluene increases the saturated fatty acid content in bacterial membranes and, thus, leads to increased membrane rigidity [29]. Possibly, an altered membrane composition influenced the secretion of metabolites in *C. coralloides*. Here, a synergistic effect of the two culture conditions in the bivariate screenings was necessary to activate the production of completely new MFs.

### 3.3. Condition Specificity and Reproducibility of Newly Detected Mass Features in Bivariate OSMAC

**Figure 3:**
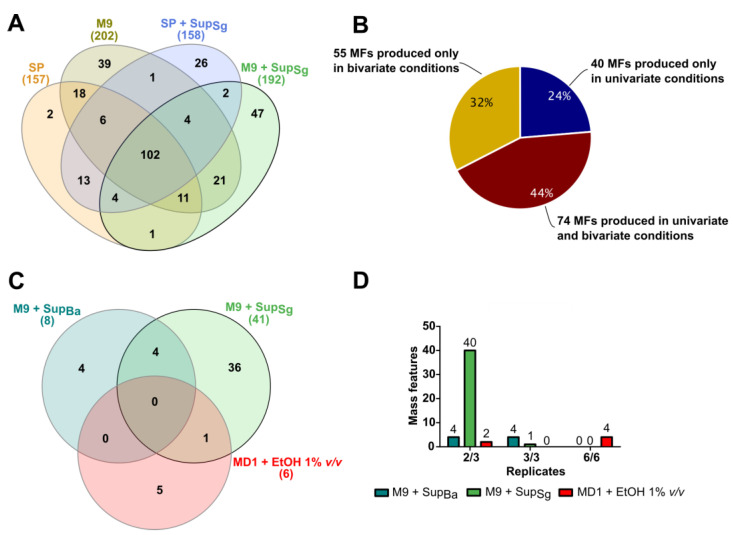
New mass features in bivariate OSMAC and the influence on their reproducibility. (**A**) The Venn diagram displays the appearance and overlap of detected MFs in the control (SP, orange), the univariate OSMAC (M9 = brown and SP + SupSg = purple) and the bivariate OSMAC (M9 + SupSg = green). The supernatant (Sup) was obtained from cultures of *S. griseochromogenes* (Sg). (**B**) The pie chart shows the distribution of detected MFs in the univariate (blue), in both (univariate and bivariate = red) and exclusively in the bivariate conditions (yellow). The Venn diagram (**C**) and the bar chart (**D**) evaluate the appearance of newly detected MFs in replicate cultivations of three bivariate OSMAC conditions: (i) M9-medium with supernatant of *B. amyloliquefaciens* (Ba) (M9 + SupBa = cyan), (ii) M9 medium with Sg supernatant (M9 + SupSg = green) and (iii) MD1 medium with the solvent ethanol (MD1 + EtOH 1% *v*/*v* = red). The appearance of newly detected MFs in bivariate OSMAC was rated with the rediscovery of MFs (i) in two of three samples in triplicate (2/3), (ii) in all three samples in triplicate (3/3) or (iii) in all samples of the two cultivated triplicates (6/6). One triplicate originated from one SD plate. Each SD plate was repeated so that in total, two triplicates of each bivariate condition were obtained.

To investigate the condition specificity of the produced MFs, we compared the detected MFs in the control, univariate and bivariate cultures. As shown in Figure 3A, we evaluated the condition specificity of the bivariate OSMAC condition with M9 medium and the supernatant additive of *S. griseochromogenes* in a Venn diagram. The MFs originating from the media themselves were already subtracted beforehand. As the reference group, SP medium was used, for which we detected 157 new MFs. The two univariate OSMAC conditions, i.e., M9 medium without an additive and SP medium with the *S. griseochromogenes* supernatant, resulted in 202 and 158 new MFs, respectively. In total, we obtained, in the bivariate OSMAC condition, 192 new MFs. In the Venn diagram, 102 MFs were shared by all four cultures, two were uniquely detected in the reference group, 39 MFs were specific to the univariate M9 culture, 26 were specific to the univariate SP medium with the *S. griseochromogenes* supernatant additive and, lastly, 47 new MFs were specific to the bivariate combination of M9 medium and the *S. griseochromogenes* supernatant additive. The analyses for the other 11 bivariate combinations and their condition-specific new MFs can be found in Figure S5. All in all, unique mass features were detected in the control condition, in the univariate condition (except for the solvent additives) and in three bivariate conditions: M9 medium with the supernatant of *B. amyloliquefaciens*, M9 medium with the supernatant of *S. griseochromogenes*, and MD1 medium with added ethanol.

A total number of 169 new MFs were detected that were specific to the univariate and bivariate conditions after the subtraction of all detected MFs in the control group (SP-medium). (Figure 3B). Among them, 24% were specific to the univariate conditions, 44% were detected in the univariate and bivariate conditions and 32% were specific to the bivariate conditions.

Our results are a strong indication that synergistic effects can result from the specific combination of multiple elicitors in one OSMAC cultivation. In this study, the specific combination of MD1-medium with 1% *v*/*v* EtOH and M9-medium with supernatants of Sg and Ba resulted in the production of new unique bivariate-specific MFs. In particular, M9-medium with the Sg supernatant showed the highest number of unique MFs (36) compared to the other conditions (Figure 3C). We hypothesized that the specific combination of these stress factors, particularly starvation, in addition to elicitors from other microbes activates multiple BGCs for survival and defense. To examine the reproducibility of the production of unique bivariateMFs, all bivariate conditions were tested twice in triplicate cultivations. A total of 44 out of 48 MFs that were only produced through the combination of M9-medium with the supernatant were only detected in two samples of the triplicates. Most unique MFs (four out of six) investigated in the bivariate condition MD1-medium with 1% *v*/*v* EtOH were detected in both triplicates (Figure 3D). This shows that the use of chemical elicitors over biotic additives results in higher reproducibility for producing new MFs in complex OSMAC cultivations conditions.

Combining specific triggers in OSMAC experiments can reliably activate the production of new SMs in *C. coralloides* and is therefore an approach that can accelerate novel bioactive compound identification during drug discovery projects.

### 3.4. Identification and Bioinformatics Assignment of Secondary Metabolites in Bivariate OSMAC

**Figure 4:**
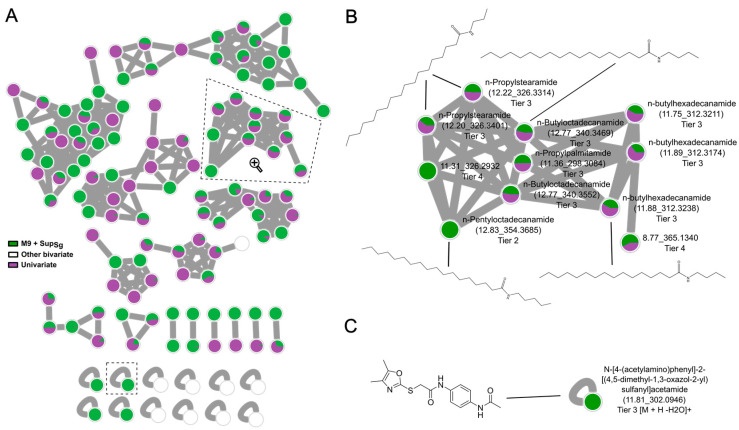
Molecular network and structural elucidation of new mass features in bivariate conditions. (**A**) Molecular network of all detected unique MFs in bivariate conditions and related MFs in univariate conditions. Each node represents a unique MF, including a pie chart with the highest observed abundance in bivariate (M9-medium with Sg supernatant = green, M9-medium with Ba supernatant and MD1-medium with 1% *v*/*v* EtOH = white) and related univariate conditions (purple). (**B**) Molecular family including possible structure and labeling of each ion with name, MFs (*m*/*z*_rt) and annotation level (tier 4 = precursor mass and a unique retention time with no further annotation, tier 3 = best hit during in silico annotation and tier 2 = GNPS online spectral library match). All *m*/*z* values represent [M + H]+ unless specified otherwise (shown with labeling). (**C**) Single ion node including possible structure and labeling.

Molecular networking is an established tool in research to analyze the LC-MS data of compounds [23]; therefore, we used this method to gain more insights into the chemical composition and structural relations of the newly produced bivariate-specific MFs. Overall, various molecular families of up to 24 nodes, some smaller families with three or two ions and single nodes were investigated (Figure 4A and Figure S6). Molecular families consist of connected nodes that embody networks of structurally related MFs. The molecular network revealed that bivariate OSMAC conditions resulted in the production of new chemical products and new chemical derivatives that were already detected in univariate conditions. Moreover, the newly generated MFs in the bivariate conditions showed *m*/*z* values between 192 and 627 within a retention time frame between 2 and 12 min (Table S7). This indicates that bivariate conditions lead to the production of various chemical structures with different properties such as polarity, size and complexity.

One MF in the cultivations in M9-medium with the supernatant of *S. griseochromogenes* resulted in an online spectral library hit, with the GNPS spectral library identifying *N*-pentyloctadecanamide (Figure S4). This molecule appears in a molecular family of 11 nodes including other related structural molecules which were also detected in univariate conditions (Figure 4B). These structures represent molecules from the family of *N*-acyl amines and play a role in the self-defense of microorganisms as fatty amides. Various naturally occurring fatty amides have been reported to exhibit biological activity, like 7-Octadecenamide, which showed antimicrobial activity against methicillin-resistant *Staphylococcus aureus* [30,31,32]. Therefore, *N*-pentyloctadecanamide represents a potential new chemical derivative specific to the bivariate condition of *N*-acyl fatty amines produced by *C. coralloides*. Further in silico fragmentation analysis of a bivariate-specific singlet node resulted in a potential chemical structure including a sulfur moiety, benzene and an oxazole substructure (Figure 4C). The potentially annotated chemical structure *N*-[4-(acetylamino)phenyl]-2-[(4,5-dimethyl-1,3-oxazol-2-yl)sulfanyl]acetamide was generated as the best hit from the in silico analysis. Furthermore, MFs investigated in a molecular family of 17 nodes showed characteristic losses of 32 from sulfur between the chemical derivatives, which might indicate possible novel sulfur containing chemical derivatives (Figure S7). While the possible sulfur-containing MFs are of high interest, not enough material in the screening plates for full structural analysis was available. Natural products with sulfur moieties, like penicillin and calicheamicin γ1, play a key role as bioactive chemical structures and provide new leads for many essential and effective drugs. Hence, sulfur-containing natural products are of interest for drug discovery, and the need for novel chemical structures with sulfur moieties from natural sources is immense [33,34,35]. The potentially annotated chemical structure class of oxazole substructures including a sulfur moiety strongly indicates the importance of using the bivariate OSMAC approach in novel drug discovery to broaden the biosynthetic production space and increase the chance of elucidating novel bioactive molecules. Moreover, known SMs produced by *C. coralloides* species containing benzene substructures are coralmycins, which showed potent antimicrobial activity against Gram-negative pathogens like *Pseudomonas aeruginosa* and *Acinetobacter baumanii* [36]. In general, oxazole-type natural products like indolyl-oxazole and oxazole alkaloids were described as promising candidates as drug leads with widespread bioactive properties like antibacterial, antifungal, anticancer and antiviral [37,38,39,40]. Our results clearly highlight the relevance of using bivariate OSMAC methods in novel bioactive natural product discovery. In this study, unique and bivariate condition-specific MFs were produced by *C. coralloides*, which represented a chemical derivative of *N*-acyl fatty amines and molecules with high potential for chemical and biological activity, including possible sulfur moiety, benzene and oxazole substructures. While interesting new MFs were generated, none of them have been related to the predicted products during genome mining (Figure 1) so far. The BGCs assigned to geosmin, VEPE/AEPE and carotenoids were detected with 100% sequence similarity. An earthy and musty smell was noticed during the cultivation of *C. coralloides* in this study, which could be attributed to geosmin production. However, detection during LC-MS/MS measurements and the annotation of geosmin were not possible, due to the volatile nature of this terpene [26]. To detect volatile natural products such as geosmin, additional GC-MS measurements must be performed [41]. No VEPE/AEPE or carotenoid was found in our study, which could be attributed to the liquid cultivation in the 24-deep-well plates. *C. coralloides* showed dispersed growth during fermentation and no formation of pellets or fruiting bodies. Because VEPE/AEPE were described to be signal molecules in the myxobacterial life cycle and *C. coralloides* are known to form orange fruiting bodies, VEPE/AEPE and carotenoids are more likely to be produced and detected during cultivation on solid surfaces where fruiting body formation is favored [13,27,42]. In addition, a myxochelin A/B BGC with 83% similarity, a myxoprincomide BGC with 66% similarity and eleven assigned BGCs with less than 50% similarity were observed during genome mining. Even though possible new MFs were detected with similarities in chemical structures such as their benzene groups, no myxochelin A/B or myxoprincomide and none of the eleven assigned BGCs could be identified. We assume that a suitable elicitor combination for the activation of the respective BCGs was not applied. Myxobacteria are predatory bacteria with a complex life cycle, including the formation of multicellular groups to travel in swarms searching for prey, produce antibacterial compounds to hunt them and aggregate into fruiting bodies as a response to starvation. The complex life of myxobacteria is highly regulated through intercellular communication and extracellular signals [42,43]. Therefore, the combination of two OSMAC conditions may not be sufficient to reflect the stress to which *C. coralloides* is exposed in a natural environment. To further increase the metabolic space of *C. coralloides* and to activate the production of assigned BGCs, the combination of other bivariate OSMAC conditions, including co-cultivation and growth on solid media, can be tested. Nevertheless, the gained results in this study strongly indicate that the combination of several elicitors in OSMAC experiments expands the biosynthetic production space by the number of chemical derivatives and potential novel SMs. Therefore, we suggest that the bivariate OSMAC design should be an essential tool in future natural product research and can lead to the identification of novel bioactive compounds.

## 4. Conclusions

Inducing SM production in wild types has been challenging due to many silenced regions in the genome under standard laboratory conditions. OSMAC screenings have been established as a simple and untargeted strategy to activate biosynthetic potential using diverse unspecific stimuli during the cultivation of microbes. However, a global picture of secondary metabolism from bacteria has not yet been described [36]. Here, we proposed a new strategy of bivariate OSMAC screenings, i.e., exposing *C. coralloides* to combined elicitors and profiting from synergistic effects in bivariate OSMAC cultivations. To demonstrate that bivariate OSMAC designs efficiently generate new and condition-specific SMs, we analyzed and compared the generated MFs in univariate and bivariate OSMAC. Our study clearly pointed out an increase in potentially interesting MFs in bivariate OSMAC compared to the corresponding univariate, or one-factor-at-a-time, approach. The high condition specificity of new MFs of over 30% and the annotation of possible novel natural molecules, including the sulfur moiety and *N*-acyl fatty amine derivative, in our bivariate OSMAC designs stresses the importance of using a bivariate approach for further expansion of the SM space for both previously described and undescribed microbes. The presented bivariate OSMAC design/approach is a first step towards the implementation of multifactorial screenings for the discovery of new natural compounds. The exposure to multiple elicitors opens an almost infinite number of combinations of stimuli to trigger the production of many compounds at the same time. The production of condition-specific MFs containing highly interesting substructures proves that bivariate OSMAC conducted in microtiter plates allows for the screening of suitable cultivation conditions for the production of new SMs and BCG activation. Further studies in scale up and further studies in isolation of the compounds are necessary to fully elucidate the compound identiy. To this end, our bivariate approach and the implementation of multifactorial OSMAC screenings are crucial to succeeding in discovering new SMs, which will eventually translate into the discovery of new natural products and, in the long run, activating silent BGCs [44].

## Data Availability

The data are contained within the article and Supplementary Materials, and the full MS/MS dataset is publicly available as a MassIVE Dataset with accession number MSV000093079.

## Supplementary Materials

The following supporting information can be downloaded at https://www.mdpi.com/article/10.3390/microorganisms11102592/s1. Table S1. Result summary of genome analysis of *C. coralloides* DSM2259; Table S2. List of chemicals, chemical formulae, and supplier; Table S3. Steps and settings used for raw data processing in MZmine 2.35; Table S4. Steps and settings used for peak list processing in MZmine 2.35; Table S5. List of control groups and background samples for each univariate experiment with *C. coralloides*; Table S6. List of background samples and univariate samples for each bivariate experiment with *C. coralloides*; Table S7. New mass features from bivariate OSMAC experiments and culture condition sets under which they were produced; Figure S1. Inoculation plan for SD plates type 1 with added cell pellets; Figure S2. Inoculation plan for SD plates types 2 with added supernatants; Figure S3. Inoculation plan for SD plates type 3 with added organic solvents; Figure S4. GNPS spectral library hit for n-Pentyloctadecanamide; Figure S5. Venn diagram of detected MFs, Figure S6. Molecular network of all detected unique MFs in bivariate conditions and related MFs in univariate conditions; Figure S7. On the left a molecular family including labeling of each ion.
