# Supplementary material for: Bivariate One Strain Many Compounds Designs Expand the Secondary Metabolite Production Space in Corallococcus coralloides
Source: Microorganisms. 2023 Oct 20;11(10):2592. doi: 10.3390/microorganisms11102592 (PMC10609524; doi:10.3390/microorganisms11102592)
Supplement: Supplementary file 1 [file microorganisms-11-02592-s001.zip › microorganisms-2650990-supplementary.pdf]

Supporting Information:

Table S1. Result summary of genome analysis of *C. coralloides* DSM2259 genome (NCBI accession # CP003389) with antiSMASH. NP = natural product, bp = base pairs, DB = database

Cluster ID	NP class	bp_{start}	bp_{end}	# bp	Predicted BGC	sequence similarity [%]	DB BGC	Origin of DB BGC
1	terpene	913,294	930,557	17,263	carotenoid	100%	BGC0000648	*Myxococcus xanthus*
2	NRPS, T1PKS	1,032,294	1,080,211	47,917	-	-	-	-
3	NRPS-like	1,827,140	1,868,365	41,225	VEPE / AEPE / TG-1	100%	BGC0000871	*Myxococcus xanthus DK 1622*
4	terpene	2,119,082	2,141,484	22,402	-	-	-	-
5	T1PKS, NRPS	2,428,410	2,482,711	54,301	chloromyxamide	13%	BGC0001945	*Myxococcus sp.*
6	T1PKS	2,606,636	2,651,986	45,350	chloromyxamide	27%	BGC0001945	*Myxococcus sp.*
7	NRPS	2,816,735	2,882,770	66,035	-	-	-	-
8	RiPP-like	3,013,876	3,022,723	8,847	-	-	-	-
9	T1PKS, NRPS-like, NRPS	3,103,725	3,257,110	153,385	-	-	-	-
10	NRPS, T1PKS	3,442,275	3,494,934	52,659	BE-43547A1 / BE-43547A2 / BE-43547B1 / BE-43547B2 / BE-43547B3 / BE-43547C1 / BE-43547C2	10%	BGC0001330	*Micromonospora sp. RV43*
11	lanthipeptide-class-i	4,030,200	4,051,486	21,286	-	-	-	-
12	NRPS, T1PKS	4,186,913	4,241,185	54,272	-	-	-	-
13	T1PKS, NRPS	4,412,963	4,485,151	72,188	-	-	-	-
14	lanthipeptide-class-i	4,529,903	4,551,241	21,338	-	-	-	-
15	RiPP-like	4,746,094	4,757,661	11,567	-	-	-	-
16	NRPS	4,857,415	4,924,895	67,480	-	-	-	-
17	lanthipeptide-class-ii	5,052,309	5,073,083	20,774	-	-	-	-
18	NRPS, T1PKS	5,209,355	5,345,413	136,058	myxoprincomide-c506	66%	BGC0000393	*Myxococcus xanthus DK 1622*

19	RRE-containing	5,384,977	5,403,935	18,958	-	-	-	-
20	NRPS, T1PKS	5,481,949	5,577,500	95,551	myxochelin A / myxochelin B	83%	BGC00001345	*Stigmatella aurantiaca Sg a15*
21	NRPS	5,625,043	5,674,634	49,591	-	-	-	-
22	RiPP-like	5,680,374	5,690,671	10,297	-	-	-	-
23	LAP,thiopeptide, RiPP-like	5,825,525	5,852,242	26,717	ficellomycin	5%	BGC0001593	*Streptomyces ficellus*
24	T1PKS	6,407,278	6,448,051	40,773	minutissamide A / minutissamide C / minutissamide D	30%	BGC0001952	*Anabaena minutissima UTEX B 1613*
25	lanthipeptide-class-i	6,566,995	6,592,859	25,864	-	-	-	-
26	terpene	6,928,334	6,945,580	17,246	-	-	-	-
27	thiopeptide, thioamitides	7,099,821	7,138,262	38,441	-	-	-	-
28	NRPS, T1PKS	7,184,802	7,246,797	61,995	nostopeptolide A2	50%	BGC0001028	*Nostoc sp. GSV224*
29	NRPS, arylpolyene	7,341,187	7,422,939	81,752	APE Ec	36%	BGC0000836	*Escherichia coli* CFT073
30	lanthipeptide-class-ii	7,528,620	7,551,841	23,221	-	-	-	-
31	butyrolactone	8,181,142	8,191,966	10,824	-	-	-	-
32	NRPS-like	8,321,728	8,364,736	43,008	myxochromide D	10%	BGC0002671	*Verrucosispora sp.*
33	phenazine	8,386,250	8,406,681	20,431	streptobactin	0.11	BGC0000368	Streptomyces sp. ATCC 700974
34	T3PKS	9,028,261	9,069,358	41,097	alkylpyrone-407 / alkylpyrone-393	0.34	BGC0001964	Cystobacterineae bacterium
35	terpene	9,239,314	9,261,572	22,258	geosmin	1	BGC0000661	Nostoc punctiforme PCC 73102
36	RiPP-like, RRE-containing	9,512,033	9,533,635	21,602	-	-	-	-

Table S2. List of chemicals, chemical formulae, and supplier. Chemicals were used in the purest form available.

Chemical	Chemical formula	Supplier	Chemical	Chemical formula	Supplier
BactoPeptone	-	Beckto Dickinson	magnesium sulfate heptahydrate	$MgSO_4$ x 7 H_2O	Carl Roth
meat extract	-	Fluka	dipotassium-phosphate	K_2HPO_4	Carl Roth
raffinose D(+) pentahydrate	$C_{18}H_{32}O_{16}$ x 5 H_2O	Sigma Aldrich	calcium chloride dihydrate	$CaCl_2$ x 2 H_2O	Carl Roth
sucrose D(+)	$C_{12}H_{22}O_{11}$	Carl Roth	vitamin B12	$C_{63}H_{88}CoN_{14}O_{14}P$	Carl Roth
galactose D(+)	$C_6H_{12}O_6$	Carl Roth	EDTA	$C_{10}H_{16}N_2O_8$	Carl Roth
soluble starch	$(C_6H_{10}O_5)n$	Carl Roth	iron (II) sulfate heptahydrate	$FeSO_4$ x 7 H_2O	Carl Roth
BactoCasitone	-	Beckto Dickinson	zinc sulfate heptahydrate	$ZnSO_4$ x 7 H_2O	AppliChem
boric Acid	H_3BO_3	Carl Roth	manganese (II) cloride tetrahydrate	$MnCl_2$ x 4 H_2O	Carl Roth
cobalt (II) chloride hexahydrate	$CoCl_2$ x 6 H_2O	Carl Roth	copper (II) chloride dihydrate	$CuCl_2$ x 2 H_2O	Carl Roth
nickel (II) chloride dihydrate	$NiCl_2$ x 6 H_2O	Alfa Aesar	sodium molybdate dihydrate	$MoNa_2O_4$ x 2 H_2O	Carl Roth
sodium chloride	NaCl	Carl Roth	D (+) glucose	$C_6H_{12}O_6$	Carl Roth
malt extract	-	Carl Roth	Bacto yeast extract	-	Beckto Dickinson
potassium dihydrogen phosphate	KH_2PO_4	Carl Roth	sodium nitrate	$NaNO_3$	Carl Roth
manganese (II) sulfate hydrate	$MnSO_4$ x H_2O	Carl Roth	thiamine-HCl	$C_{12}H_{17}ClN_4OS$ x HCl	Carl Roth
ethyl acetate	$C_4H_8O_2$	VWR	L-proline	$C_5H_9NO_2$	Carl Roth
ethanol	C_2H_6O	Carl Roth	disodium hydrogen phosphate	Na_2HPO_4 x 2 H_2O	AppliChem
toluene	C_7H_8	Fisher Scientific	ammonium chloride	NH_4Cl	Carl Roth
methanol	CH_4O	Carl Roth			

Table S3. Steps and settings used for raw data processing in MZmine 2.35.

Input	Step	Settings	Output
raw data, mzXML-format	Peak Detection -> Mass Detection	MS level: 1<br>Mass Detector: Centroid<br>Noise level: 1.0E3	mass list
raw data, mzXML-format	Peak Detection -> Mass Detection	MS level: 2<br>Mass Detector: Centroid<br>Noise level: 1.0E1	mass list
mass list	Peak Detection -> Chromatogram Builder	MS level: 1<br>Min time span: 0.01 min<br>Min height: 1.0E3<br>m/z tolerance: 0.01 m/z or 0 ppm	peak lists, suffix: chromatograms

Table S4. Steps and settings used for peak list processing in MZmine 2.35.

Input	Step	Settings	Output
peak lists, suffix: chromatograms	Peak Detection -> Chromatogram Deconvolution	Algorithm: Baseline cut-off with Min peak height: 1.5E3, Peak duration range (min): 0.01 – 3.00, Baseline level: 1.0E3<br>m/z range for MS2 scan paring (Da): 0.01<br>RT range for MS2 scan pairing (min): 0.1	peak lists, suffix: chromatograms deconvoluted
peak lists, suffix: chromatograms deconvoluted	Isotopes -> Isotopic peaks grouper	m/z tolerance: 0.01 m/z or 0 ppm<br>RT tolerance: 0.1 min<br>Maximum charge: 3<br>Representative isotope: Most intense	peak lists, suffix: chromatograms deconvoluted deisotoped
peak lists, suffix: chromatograms deconvoluted deisotoped	Alignment -> Join Aligner	m/z tolerance: 0.01 m/z or 0 ppm<br>Weight for m/z: 80<br>RT tolerance: 0.1 min<br>Weight for RT: 20	aligned peak list
aligned peak list	Filtering -> Peak list rows filter	Minimum peaks in a row: 2<br>Keep only peaks with MS2 scan	aligned peak list, suffix: filtered
aligned peak list, suffix:<br>gap-filled	Gap-filling -> Peak finder	Intensity tolerance: 10%<br>m/z tolerance: 0.01 m/z or 0 ppm<br>Retention time tolerance: 0.2 absolute (min)	aligned peak list, suffix: filtered gap-filled

Table S5. List of control groups and background samples for each univariate experiment with *C. coralloides*. The control group for all univariate experiments was *C. coralloides* grown on SP medium without any additives.

Univariate Experiments	Background samples to be subtracted
SP medium + P_{Ba}	SP medium and extracted P_{Ba}
SP medium + P_{Sg}	SP medium and extracted P_{Sg}
SP medium + Sup_{Ba}	SP medium and extracted Sup_{Ba}
SP medium + Sup_{Sg}	SP medium and extracted Sup_{Sg}
SP medium + 1% v/v Tol	SP medium with 1% v/v toluene
SP medium + 1% v/v EtOH	SP medium with 1% v/v ethanol
M9 medium	M9 medium
MD1 medium	MD1 medium

Table S6. List of background samples and univariate samples for each bivariate experiment with *C. coralloides* (Cc). The control group for all bivariate experiments was *C. coralloides* grown on SP medium without any additives. A combined background sample was used for the experiments with organic solvents, i.e., the solvent (ethanol (EtOH) or toluene (Tol)) was directly added to the respective medium which was then extracted.

**Bivariate Experiments**	**Background samples to be subtracted**	**Univariate condition 1: additive (to be subtracted)**	**Univariate condition 2: medium (to be subtracted)**
M9 medium + P_{Sg}	M9 medium and extracted P_{Sg}	Cc on SP medium + P_{Sg}	Cc on M9 medium
M9 medium + P_{Ba}	M9 medium and extracted P_{Ba}	Cc on SP medium + P_{Ba}	Cc on M9 medium
MD1 medium + P_{Sg}	MD1 medium and extracted P_{Sg}	Cc on SP medium + P_{Sg}	Cc on MD1 medium
MD1 medium + P_{Ba}	MD1 medium and extracted P_{Ba}	Cc on SP medium + P_{Ba}	Cc on MD1 medium
M9 medium + Sup_{Sg}	M9 medium and extracted Sup_{Sg}	Cc on SP medium + Sup_{Sg}	Cc on M9 medium
M9 medium + Sup_{Ba}	M9 medium and extracted Sup_{Ba}	Cc on SP medium + Sup_{Ba}	Cc on M9 medium
MD1 medium + Sup_{Sg}	MD1 medium and extracted Sup_{Sg}	Cc on SP medium + Sup_{Sg}	Cc on MD1 medium
MD1 medium + Sup_{Ba}	MD1 medium and extracted Sup_{Ba}	Cc on SP medium + Sup_{Ba}	Cc on MD1 medium
M9 medium + 1% v/v Tol	M9 medium with 1% v/v Tol	Cc on SP medium + 1% v/v Tol	Cc on M9 medium
M9 medium + 1% v/v EtOH	M9 medium with 1% v/v EtOH	Cc on SP medium + 1% v/v EtOH	Cc on M9 medium
MD1 medium + 1% v/v Tol	MD1 medium with 1% v/v Tol	Cc on SP medium + 1% v/v Tol	Cc on MD1 medium
MD1 medium + 1% v/v EtOH	MD1 medium with 1% v/v EtOH	Cc on SP medium + 1% v/v EtOH	Cc on MD1 medium

Table S7. New mass features from bivariate OSMAC experiments and culture condition sets under which they were produced. P_{Ba} = B. amyloliquefaciens pellet, Sup_{Ba} = B. amyloliquefaciens supernatant, P_{Sg} = S. griseochromogenes pellet, Sup_{Sg} = S. griseochromogenes supernatant, EtOH = Ethanol, Tol = toluene. All m/z represent [M+H]+ unless specified otherwise. n/a = not applicable

Mass feature [t_R _m/z]	t_R [min]	m/z [-]	Producing conditions	Neutral sum formula
2.3_298.0922 [M+H] 2+	2.3	298.0922	M9 + SupSg	C22H22N14O5S
2.3_282.1064	2.3	282.1064	M9 + SupSg	n/a
2.38_595.1692	2.38	595.1692	M9 + SupSg	C22H22N14O5S
2.47_314.0763 [M+H] 2+	2.47	314.0763	M9 + SupSg	C22H22N14O5S2
2.48_627.1379	2.48	627.1379	M9 + SupSg	C22H22N14O5S2
2.48_282.1076	2.48	282.1076	M9 + SupSg	n/a
2.49_213.1	2.49	213.1	MD1 + EtOH 1 % v/v	C8H12N4O3
2.59_415.1306	2.59	415.1306	M9 + SupSg	C13H18N8O8
2.66_297.0738 [M+H-H2O]+	2.66	297.0738	M9 + SupSg	n/a
3.82_395.1069	3.82	395.1069	M9 + SupSg, M9 + SupBa	C15H18N6O5S
4.59_293.093	4.59	293.093	MD1 + EtOH 1 % v/v	C11H18NO6S
5.11_240.0653	5.11	240.0653	MD1 + EtOH 1 % v/v	C6H13N3O5S
5.11_192.0625	5.11	192.0625	MD1 + EtOH 1 % v/v	C7H11O6
7.48_385.2918	7.48	385.2918	M9 + SupBa	C22H40O5
7.54_341.2658	7.54	341.2658	M9 + SupBa	C20H36O4
7.99_443.3359	7.99	443.3359	M9 + SupBa	C25H46O6
8.12_399.3067	8.12	399.3067	M9 + SupBa	C23H42O5
8.38_326.3675	8.38	326.3675	M9 + SupSg, MD1 + EtOH 1 % v/v	C21H45N2
8.96_427.3841	8.96	427.3841	MD1 + EtOH 1 % v/v	C16H46N10O3
10.19_421.337	10.19	421.337	M9 + SupSg	C19H44N6O2S
10.24_377.3127	10.24	377.3127	M9 + SupSg	C18H40N4O4
10.48_489.3049	10.48	489.3049	M9 + SupSg	n/a
10.5_589.4044	10.5	589.4044	M9 + SupSg	C23H56N8O9
10.55_545.3787	10.55	545.3787	M9 + SupSg	C37H52O3
10.55_268.2612	10.55	268.2612	M9 + SupSg	C10H31N6O2
10.55_268.2548	10.55	268.2548	M9 + SupSg	C10H31N6O2
10.6_501.3551	10.6	501.3551	M9 + SupSg	C31H48O5
10.94_603.4183	10.94	603.4183	M9 + SupSg	C23H50N14O5
11.04_559.3937	11.04	559.3937	M9 + SupSg, M9 + SupBa	C15H46N18O5
11.08_493.3903	11.08	493.3903	M9 + SupSg	C20H48N10O4
11.1_515.3706	11.1	515.3706	M9 + SupSg, M9 + SupBa	C17H46N12O6
11.17_471.3465	11.17	471.3465	M9 + SupSg	C15H42N12O5
11.18_302.0946 [M+H-H2O]+	11.18	302.0946	M9 + SupSg	C15H15N3O2S
11.21_449.3645	11.21	449.3645	M9 + SupSg	C18H44N10O3
11.25_617.434	11.25	617.434	M9 + SupSg	C28H56N8O7

11.31_405.3414	11.31	405.3414	M9 + SupSg, M9 + SupBa	C16H40N10O2
11.31_326.2932	11.31	326.2932	M9 + SupSg	C15H37N5O
11.4_573.4102	11.4	573.4102	M9 + SupSg	C31H52N6O4
11.66_485.3625	11.66	485.3625	M9 + SupSg	C27H44N6O2
11.68_463.3811	11.68	463.3811	M9 + SupSg	C24H52N3O3S
11.78_609.4661	11.78	609.4661	M9 + SupSg	C33H62N5O3S
11.85_587.4242	11.85	587.4242	M9 + SupSg	C25H60N7O4S2
11.86_565.4442	11.86	565.4442	M9 + SupSg	C29H62N3O7
11.87_375.3335	11.87	375.3335	M9 + SupSg	C17H40N7O2
11.98_521.4198	11.98	521.4198	M9 + SupSg	C13H48N18O4
12.05_477.3957	12.05	477.3957	M9 + SupSg	C26H48N6O2
12.2_433.3717	12.2	433.3717	M9 + SupSg	C22H46N4O3
12.38_535.4346	12.38	535.4346	M9 + SupSg	C12H48N21O3
12.66_350.3286	12.66	350.3286	M9 + SupSg	C17H41N4O3
12.83_354.3685	12.83	354.3685	M9 + SupSg	C23H47NO

	1	2	3	4	5	6
A	Control:<br>75 µL preculture *C. coralloides*<br>+<br>2925 µL SP medium			Blank<br>3000 µL<br>SP	Minimal medium:<br>75 µL preculture Cc<br>+<br>2925 µL MD1 medium	
B	Additive 1:<br>75 µL preculture Cc +<br>2825 µL SP medium +<br>100 µL pellet Ba		Additive 2:<br>75 µL preculture Cc +<br>2825 µL SP medium +<br>100 µL pellet Sg		Complex medium:<br>75 µL preculture Cc<br>+<br>2925 µL M9 medium	
C	Complex medium + Additive 1:<br>75 µL preculture Cc +<br>2825 µL MD1 medium +<br>100 µL pellet Ba			Complex medium + Additive 2:<br>75 µL preculture Cc +<br>2825 µL MD1 medium +<br>100 µL pellet Sg		
D	Minimal medium + Additive 1:<br>75 µL preculture Cc +<br>2825 µL M9 medium +<br>100 µL pellet Ba			Minimal medium + Additive 2:<br>75 µL preculture Cc +<br>2825 µL M9 medium +<br>100 µL pellet Sg		

Figure S1. Inoculation plan for SD plates type 1 with added cell pellets. White = control and blank, grey = univariate experiments, black = bivariate experiments.

	1	2	3	4	5	6
A	Control:<br>75 µL preculture *C. coralloides*<br>+<br>2925 µL SP medium			Blank<br>3000 µL<br>SP	Minimal medium:<br>75 µL preculture Cc<br>+<br>2925 µL MD1 medium	
B	Additive 1:<br>75 µL preculture Cc +<br>2625 µL SP medium +<br>300 µL supernatant Ba		Additive 2:<br>75 µL preculture Cc +<br>2625 µL SP medium +<br>300 µL supernatant Sg		Complex medium:<br>75 µL preculture Cc<br>+<br>2925 µL M9 medium	
C	Complex medium + Additive 1:<br>75 µL preculture Cc +<br>2625 µL MD1 medium +<br>300 µL supernatant Ba			Complex medium + Additive 2:<br>75 µL preculture Cc +<br>2625 µL MD1 medium +<br>300 µL supernatant Sg		
D	Minimal medium + Additive 1:<br>75 µL preculture Cc +<br>2625 µL M9 medium +<br>300 µL supernatant Ba			Minimal medium + Additive 2:<br>75 µL preculture Cc +<br>2625 µL M9 medium +<br>300 µL supernatant Sg		

Figure S2. Inoculation plan for SD plates types 2 with added supernatants. White = control and blank, grey = univariate experiments, black = bivariate experiments.

<table>
<tr><th></th><th>1</th><th>2</th><th>3</th><th>4</th><th>5</th><th>6</th></tr>
<tr><td>A</td><td colspan="3">Control:
75 µL preculture C. coralloides
+
2925 µL SP medium</td><td>Blank
3000 µL
SP</td><td colspan="2">Minimal medium:
75 µL preculture Cc
+
2925 µL MD1 medium</td></tr>
<tr><td>B</td><td colspan="2">Additive 1:
75 µL preculture Cc +
2895 µL SP medium +
30 µL ethanol</td><td colspan="2">Additive 2:
75 µL preculture Cc +
2895 µL SP medium +
30 µL toluene</td><td colspan="2">Complex medium:
75 µL preculture Cc
+
2925 µL M9 medium</td></tr>
<tr><td>C</td><td colspan="3">Complex medium + Additive 1:
75 µL preculture Cc +
2895 µL MD1 medium +
30 µL ethanol</td><td colspan="3">Complex medium + Additive 2:
75 µL preculture Cc +
2895 µL MD1 medium +
100 µL toluene</td></tr>
<tr><td>D</td><td colspan="3">Minimal medium + Additive 1:
75 µL preculture Cc +
2895 µL M9 medium +
30 µL ethanol</td><td colspan="3">Minimal medium + Additive 2:
75 µL preculture Cc +
2895 µL M9 medium +
30 µL toluene</td></tr>
</table>

Figure S3. Inoculation plan for SD plates type 3 with added organic solvents. White = control and blank, grey = univariate experiments, black = bivariate experiments.

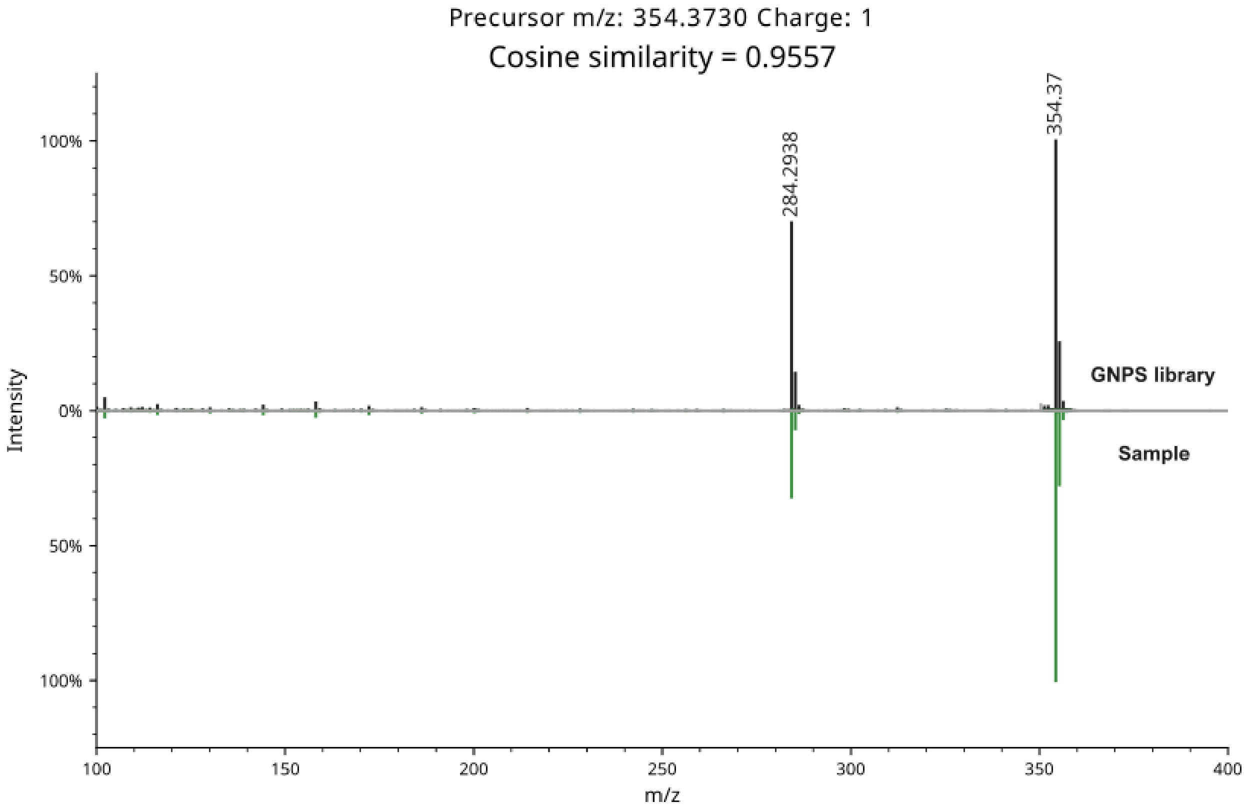

Figure S4. GNPS spectral library hit for n-Pentyloctadecanamide. On top the MSMS fragmentation pattern from the spectral library and on the bottom the MSMS acquired in this study.

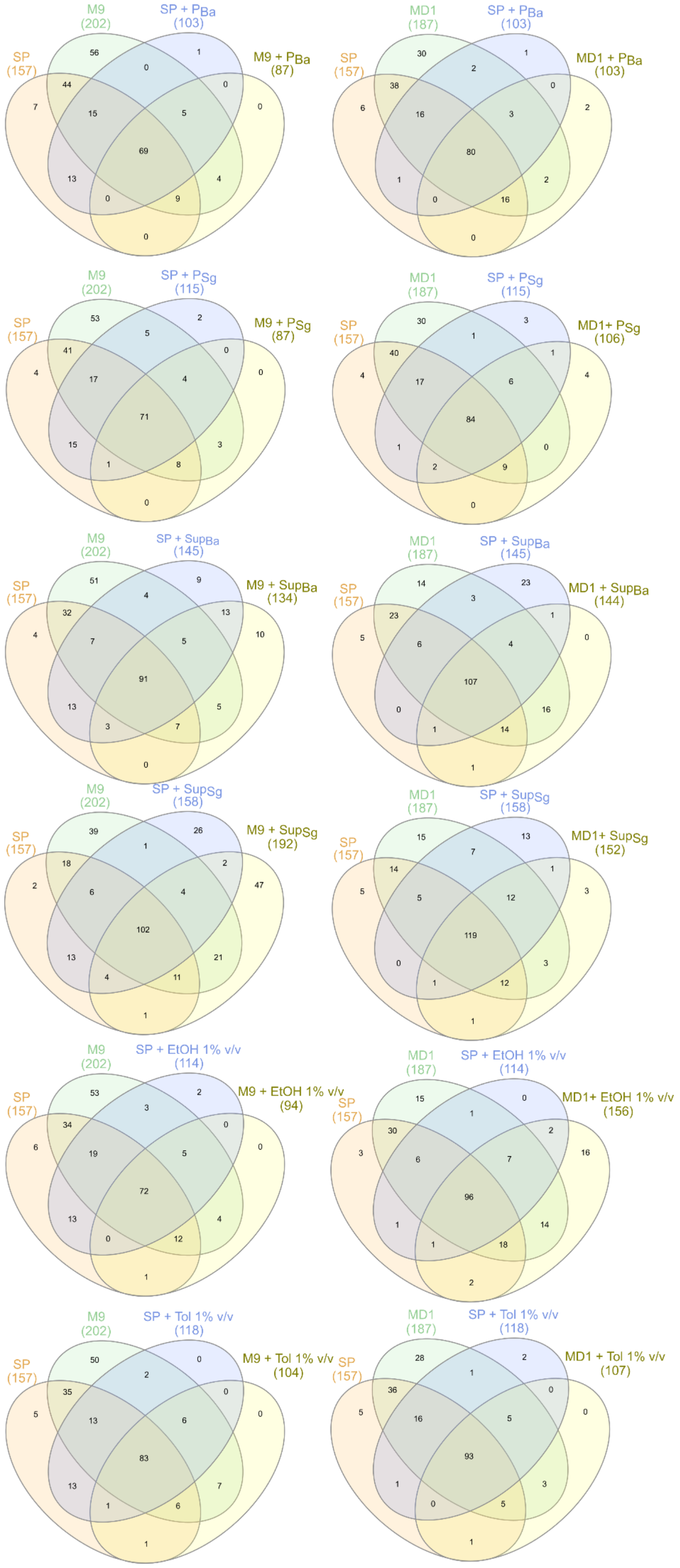

Figure S5. Venn diagram of detected MFs in the control (SP, orange), all univariate conditions (minimal medium = green with M9 on the right and MD1 on the left, biotic and chemical additives = blue) and all bivariate conditions (yellow).

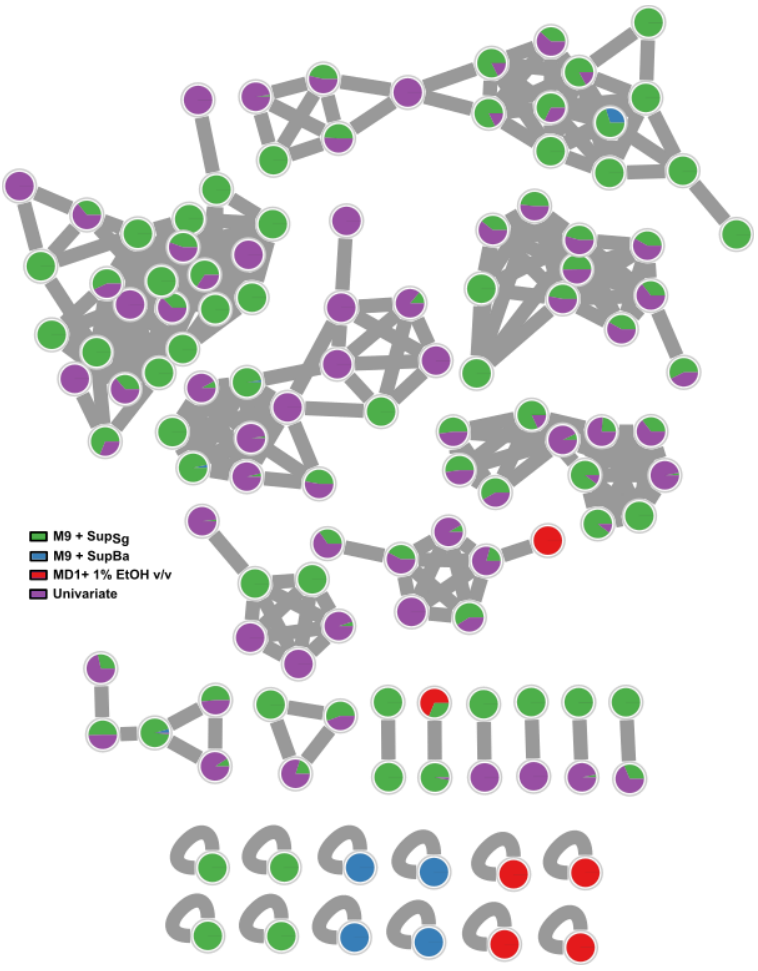

Figure S6. Molecular network of all detected unique MFs in bivariate conditions and related MFs in univariate conditions. Each nodes represents a unique MFs including a pie chart with the highest observed abundance in bivariate (M9-medium with Sg supernatant = green, M9-medium with Ba supernatant = blue and MD1-medium with 1 % v/v EtOH = red) and related univariate conditions (purple).

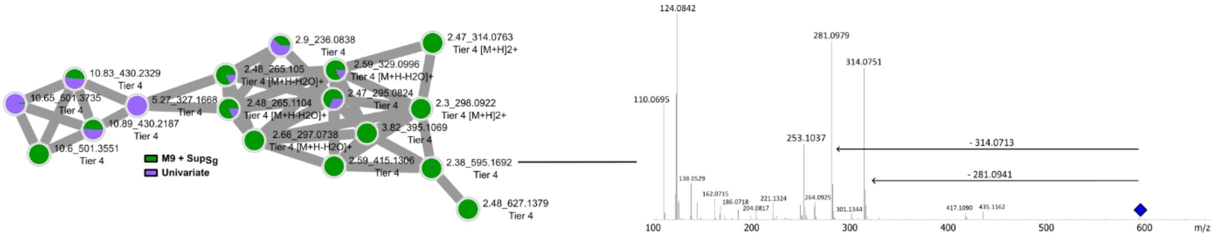

Figure S7. On the left a molecular family including labeling of each ion with Name, MFs (m/z_rt) and annotation level (tier 4 = precursor mass and a unique retention time with no further annotation). All m/z represent [M + H]+ unless specified otherwise (shown with labeling). Each nodes represents a unique MFs including a pie chart with the highest observed abundance in bivariate (M9-medium with Sg supernatant = green) and related univariate conditions (purple). On the right a fragmentation spectra of the MF 2.38_595.1692.
